# Pollution Characteristics and Health Risk Assessment of Fluoride and Potentially Toxic Elements in Coal Mine Water of Shanxi Province, North China: A Comparative Analysis of Raw Mine Water and Mine Drainage

**DOI:** 10.3390/toxics14070553

**Published:** 2026-06-25

**Authors:** Yulu Pei, Jie Luo, Chunyu Ma, Pingchuan Ma, Xin Lin, Weihua Li, Juping Yan, Xuejun Sun

**Affiliations:** 1 School of Environmental and Resource Sciences, Shanxi University, Taiyuan 030006, China; 2 School of Environmental and Resource, Taiyuan University of Science and Technology, Taiyuan 030024, China; 3 Shanxi Key Laboratory of Coordinated Management and Control for Environmental Quality, Taiyuan University of Science and Technology, Taiyuan 030024, China; 4 Shanxi Key Laboratory of Ecological Restoration for Loess Plateau, Shanxi University, Taiyuan 030006, China

**Keywords:** coal mine water, fluoride, potentially toxic elements, health risk assessment

## Abstract

Coal mining critically affects Shanxi’s economy and national energy security in China, whereas mine water significantly influences regional water quality and ecological stability. However, studies on pollution characteristics and health risks of fluoride and potentially toxic elements (PTEs) remain limited, especially comparative analyses between raw mine water and treated mine drainage. This study comprehensively analyzed the pollution characteristics of fluoride and PTEs, along with water quality evaluation, ecological risks, and human health risks associated with raw mine water and mine drainage. Fluoride concentrations in raw mine water from several mines exceeded the WHO guideline limit of 1.5 mg/L, whereas those in mine drainage were below the WHO standard. The total hazard index (THI) of fluoride in both water types was unacceptable (THI > 1). For PTEs, only arsenic in raw mine water exceeded the Grade III groundwater standard, while all PTEs in mine drainage met standards. Total health risk of PTEs in raw water was approximately one order of magnitude higher than in mine drainage, and both exceeded acceptable levels, mainly contributed by carcinogenic elements, particularly arsenic. These results underscore continuous monitoring and targeted control of arsenic are still required for safe utilization of coal mine water.

## 1. Introduction

Mine water refers to various water inflows generated during the extraction of mineral resources [1], including raw mine water and mine drainage [2]. It usually contains a variety of contaminants [3], such as excessive fluoride (F) [4] and PTEs, which directly affect its widespread use [5,6]. F^−^ is an important component of natural water [7] and the World Health Organization (WHO) recommends an acceptable F^−^ concentration of 1.50 mg/L in drinking water [8]. Excess fluoride in water causes health hazards to the natural environment [9]. PTEs are non-biodegradable pollutants that enter the water and sediment phases of aquatic ecosystems and accumulate in organisms [10].The uncontrolled discharge of coal mine water poses substantial risks of potentially toxic elements contamination to proximal aquatic systems, soil and plants [11,12,13,14]. Therefore, evaluating the mine water quality is significant to the sustainable development of water resources in China [15,16].

Coal has remained the dominant energy source in China [17], China is currently the largest coal-producing country in the world [18], with annual production nearly 3.36 billion tons in 2016 [2]. At present, domestic and international research on raw mine water and mine drainage mainly focuses on the source and mechanism analysis of PTE pollution, as well as investigations into water quality evolution and treatment technologies. For example, most investigations have concentrated on the sources of PTE contamination in surface water [19], groundwater [5,20], and sediments [21] adjacent to mining areas. Meanwhile, several other studies have explored the application of synthetic materials for the removal of PTEs from water samples [6,22,23,24,25], considering both metal species and concentrations [26,27,28,29]. Generally, raw mine water are unfit for directly used, but mine drainage, rainfall, and infiltration cause PTE enrichment, adversely affecting the groundwater and harming human health [6]. Researchers investigated the environmental pollution and human health risks of surface water samples collected from coal mines in Sunan Coal Mine, and the results revealed that chromium is the major carcinogenic factor in the study area, contributed to 95.45% of the total health risk [6], and authorities in this region must closely monitor three PTE—Fe, Mn, and Cr [6]. Therefore, conducting assessments on pollution status and human health risks of raw mine water and mine drainage can provide fundamental data support for local environmental supervision, and has a significance for the protcetion of aquatic environment surrounding mining areas, especially for the adjacent river basins.

As one of China’s key coal-producing regions [30], Shanxi Province reported a total treated mine water volume of 21.5 billion cubic meters from its 589 coal mines in 2021, corresponding to a utilization rate of 68%. The Zhuozhang River is one of the headwaters of the Haihe River Basin [31], originating in Changzhi City, Shanxi Province. In recent year, active coal mining operations along the Zhuozhang River was appeared, and anthropogenic activities possible accelerated the pollution status of the river [32,33]. However, the pollution and health risk of PTEs of coal mine water along the Zhuozhang river during coal exploration and other processes in mining areas was still limited. To better understand mine water quality characteristics and the difference between raw mine water and mine drainage, this study investigated the environmental quality and health risks of fluoride and PTEs for raw and mine drainage from five mining areas in the upper reaches of the Zhuozhang River, providing scientific recommendations for regional water conservation and water security.

## 2. Materials and Methods

### 2.1. Study Area

Zhuozhang River is an important water system in Shanxi Province and runs through two cities—Jinzhong and Changzhi—and thirteen counties [34]. The sampling sites lies within the core area of the Qinshui Coalfield, where stratigraphic units—including the Ordovician, Carboniferous, Permian, Triassic, and Cenozoic (Neogene and Quaternary) systems—are arranged from east to west in order of increasing geologic age [35]. These areas is a major coal-producing region, with coal-bearing areas covering approximately 829.7 km (71.7% of the county’s total area). The county has 7.58 billion tons of proven coal reserves and an approved annual production capacity of 16.1 million tons, hosting multiple coal mining enterprises.

**Figure 1:**
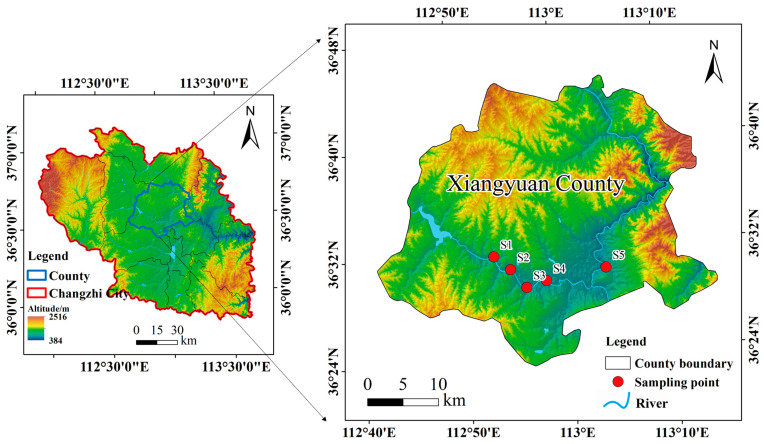
Study area and sampling points.

According to the distribution of mining areas along the western tributary of the Zhuozhang River, sampling was conducted at mines S1, S2, S3, S4, and S5 (Figure 1). For mines S1, S2, and S3, both raw water (RW) and effluent water (EW) were collected, while only effluent water was sampled for mines S4 and S5. The raw-versus-effluent comparison samples with differnet treatment method (reverse osmosis treatment and chemical treatment) were collected from S1. All the samples were collected in August 2024.

### 2.2. Analytical Methods

The pH of water samples was measured on-site using a portable water quality analyzer (Multi 3430, WTW, Weilheim, Germany), with sampling locations recorded via GPS coordinates. Water samples for PTE analysis were acidified to pH < 2 using high-purity nitric acid (HNO_3_, Chuandong Chemical Group, Chongqing, China) and stored in HDPE bottles at 4 °C. For mercury (Hg) analysis, water samples were spiked with 250 mL of BV-III grade (CMOS) HCl (Beihua Chemical, Beijing, China). Water samples for ion analysis were filtered in the field through 0.22 μm MCE membranes (25 mm × 0.22 μm, BKM Holdings, Shanghai, China).The concentrations of metal elements (Cu, Pb, As, Zn, Cd) were determined using inductively coupled plasma mass spectrometry (ICP-MS). A precisely measured 10 mL aliquot of the homogenized mine water sample was transferred into a microwave digestion vessel. Subsequently, 5 mL of concentrated nitric acid and 2 mL of hydrogen peroxide were added. The mixture was subjected to digestion using a microwave digestion system. A method blank, in which the sample was replaced with ultrapure water, was processed in parallel through the entire sample preparation procedure to monitor potential contamination throughout the process. Following digestion, a clear and transparent digestate was obtained for instrumental analysis. All samples were analyzed in duplicate to ensure the relative standard deviation (RSD) remained within 10%. Spiked samples were periodically analyzed throughout the testing sequence to verify recovery rates and confirm instrument stability. The measured concentrations in the blank samples were consistently near background levels, confirming the absence of significant contamination.

Mercury (Hg) was analyzed using a Tekran 2600 mercury analyzer (Tekran Instruments Corporation, Toronto, ON, Canada) based on the BrCl oxidation-SnCl_2_ reduction-gold amalgamation-cold vapor atomic fluorescence spectrometry (CVAFS) method. To control for data quality, a standard solution with a Hg concentration of 5 ng/L was added, and the results showed that the recovery rate was between 95% and 105%. 20% of the total number of samples was added as the instrument blank, and the resulting value was <0.1 ng/L. The field blank sample levels were also tested at the same time, and their values were also <0.1 ng/L. Fluoride (F^−^) were measured by ion chromatography with a method detection limit of 0.001 mg·L^−1^.

### 2.3. Environmental Risk Assessment Methods for PTES

#### 2.3.1. Single Factor Index Method

The Single Factor Index Method is the simplest and most commonly used approach for evaluating PTE pollution [36]. It provides an intuitive preliminary assessment of water quality by calculating a single pollution index, expressed by the formula:

(1) $$P_{i}=\frac{C_{i}}{S_{i}}\;\;$$

In the formula, $P_{i}$ is the pollution index of PTEs (i), and $C_{i}$ represents the measured concentration of PTEs (i) [37]; $S_{i}$ represents the Class III standard value for PTEs (i) in the Groundwater Environmental Quality Standards (GB/T 14848-2017) [38].

When $P_{i}$ ≤ 1, the water quality meets the Class III water quality standard, and $P_{i}$ > 1, the water quality does not meet the Class III water quality standard.

#### 2.3.2. Nemerow Index Method

The Nemerow pollution index method is a comprehensive evaluation approach based on the single factor index method [6,36]. It considers extreme values and emphasizes the most heavily polluted factor to provide an integrated assessment of various water quality parameters. The calculation formula is as follows:

(2) $$P_{N}=\sqrt{\frac{{\left(\bar{p}\right)}^{2}+{\left(P_{imax}\right)}^{2}}{2}}$$

(3) $$\bar{p}=\frac{1}{n}\sum_{i=1}^{n}P_{i}$$

In Equation (2), $P_{N}$ is Nemerow comprehensive pollution index (CPI), $P_{imax}$ represents is the maximum value of the individual pollution index of the PTE, and $\bar{p}\;$ is the average value of the individual factor index, which can be calculated from Equation (3). The grading standard for environmental quality evaluation by the single factor index method and the Nemerow pollution index method are shown in Table S1 [39].

#### 2.3.3. Human Health Risk Assessment

The non-carcinogenic health risk caused by coal mine water fluoride exposure to the populace was evaluated by the daily intake of fluoride with the tolerable threshold limit. Based on the method reported by Chicas, et al. [40], the human health risk caused by the exposure of fluoride due to oral ingestion and dermal contact for different aged people was assessed. The chronic daily intake (CDI) can be calculated as shown in Equation (4) which is the health threat through oral ingestion of fluoride.

(4) $$CDI=\frac{C\times IR\times EF\times ED}{BW\times AT}$$

where CDI is the chronic daily intake (mg/kg/day), C is the concentration of fluoride (mg/L); IR is the Ingestion Rate (L/day); EF is the Exposure Frequency; ED is the Exposure Duration (years) for oral and dermal pathways; BW is the Body Weight (kg); AT (in days) is the Average lifetime of the effects. All the factors in the equation were presented in Table S2.

The Hazard Quotient for oral ingestion (HQO) is calculated from Equation (5). The reference dosage for the oral ingestion of fluoride ${R_{f}D}_{i}$ is equal to 0.06 mg/kg-body weight/day.

(5) $$HQO=\frac{CDI}{{R_{f}D}_{i}}$$

The Total Hazard Index (THI) was calculated from Equation (11). The Hazard Quotient through the dermal contact (HQD) as shown in Equation (10) can be calculated from Equation (6) using Equations (6)–(10) along with the value of ${R_{f}D}_{d}$ which is equal to 5.82 × 10^−2^ mg/kg-bodyweight/day [40]

(6) $$CDD=\frac{DA\times EV\times SA\times EF\times ED}{BW\times AT}$$

(7) DA=K×C×t×CF

(8) $$SA=239\times H^{0.417}\times {BW}^{0.517}$$

(9) $${R_{f}D}_{d}={R_{f}D}_{i}\times {ABS}_{gi}$$

(10) $$HQD=\frac{CDD}{{R_{f}D}_{d}}$$

(11) THI=HQO+HQD

where CDD is the chronic dose via dermal pathway (mg/kg/day); DA is the Exposure dosage of every single event (mg/cm^2^/day); SA is the Skin Surface Area (cm^2^); C is the fluoride concentration (mg/L); K is the Coefficient of skin permeability; t is the contact time (h); CF is the Conversion Factor; H denotes the Average Height in cm; ${ABS}_{gi}$ is called as the Absorption factor (for all contaminants) which is equal to 1; EV (equal to 1) is the dermal contact frequency limit (L/day) [41]. All the factors in the equation were presented in Table S2.

The United States Environmental Protection Agency (USEPA) developed the Health Risk Assessment Model (HRSM) to evaluate the health risks posed by PTEs [42,43]. Two exposure pathways are considered: ingestion via oral and dermal contact [5,44]. For the same PTE, key parameters including exposure dose, carcinogenic potency factor, and reference dose differ significantly between the oral ingestion pathway and the dermal contact pathway [45,46,47]. Detailed calculation procedures are shown in Text S1.

## 3. Results and Discussion

### 3.1. Fluoride Ion Distribution Characteristics of Mine Water and Health Risk Assessment

The F^−^ concentrations in the raw water from Mine S1 (3.6 mg/L) and Mine S3 (4.4 mg/L) exceed the Chinese agricultural irrigation standards (GB 5084-2021) [48], which cannot directly used for irrigation. However, after reverse osmosis treatment, the F^−^ concentrations of raw water from Mine S1 (0.28 mg/L) meet China’s agricultural irrigation standards. The effluent water from coal Mines has F^−^ concentrations below the WHO recommended range (1.5 mg/L) for drinking [49], but higher the maximum permitted F^−^ concentration in drinking water (1.00 mg/L) in China, which indicated the effluent water unsuitable for oral ingestion [15].

Compared with previous studies, the F^−^ concentration in coal mine effluent in this study (0.38–1.4 mg/L, average 1.04 mg/L) is close to that in India (0.1–2.3 mg/L, average 1 mg/L) [50] but lower than that in Shaanxi coal mines (0.16–12.75 mg/L, average 6.1 mg/L) [51], indicating regional differences in the spatial distribution and hydrogeochemical processes of fluoride in coal mine water. Numerous studies indicate that the enrichment and depletion of F^−^ are closely related to the hydrogeochemical and alkaline conditions of groundwater [52,53]. In this study, except for the drainage water from mine S3 and S4, F^−^ concentration in mine water is exceeded the maximum permitted value (1 mg/L), and the pH of the mine water was in the range of 6.54 to 8.38, indicating that weakly alkaline conditions possibly facilitate fluoride dissolution and migration [54]. The possible reason is that under alkaline conditions, the concentration of hydroxide ions is relatively high, reducing the activity of Ca^2+^ and inhibiting the formation of CaF. Meanwhile, F^−^ in fluorine-bearing minerals is prone to ion exchange and fluoride primarily exists as free F^−^ ions [55].

**Figure 2:**
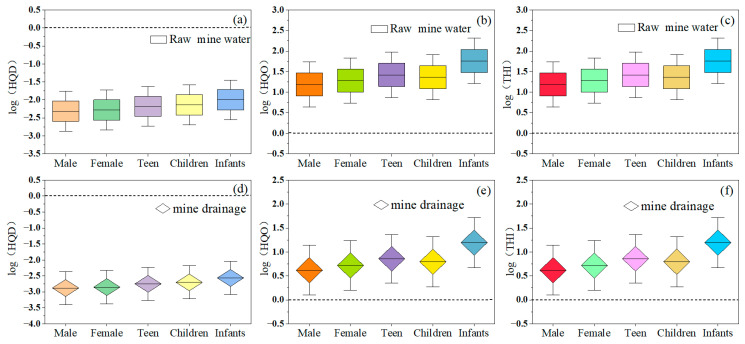
The logarithm of HQD, HQO and total hazard index (THI) values of F^−^ in raw water (**a**–**c**) and effluent water (**d**–**f**) from coal mine. Note: The dotted line represents the position where log(HQD), log(HQO), and log(THI) equal zero.

As shown in Figure 2a,d, the log (HQD) in raw water and effluent water was both lower than zero, which means that the health risk of F^−^ from oral ingestion is negligible for different age of groups. However, for adult females, adult males, teenagers, children and infants, the log (HQO) values in raw water ranged from 0.87 to 1.39 (mean: 1.19), 0.96 to 1.49 (1.28), 1.1 to 1.63 (1.42), 1.04 to 1.57 (1.36), and 1.44 to 1.97 (1.76), respectively. In Figure 2b,e, the log (HQO) in effluent water showed a similar trend to that in raw water. These results reveals that the health risk of F^−^ from dermal contact should not be ignored in different age groups.

It is worth mention that the log(THI) in raw water and effluent water was both higher than zero (Figure 2c,f), which reveals that the total risk posed by the F^−^ to the human health is at unacceptable level. Among the five categories, the average value of log (THI) in raw water are in the following order: Infants (1.76) > Teenagers (1.42) > Children (1.36) > female (1.28) > male (1.19). A comparison of log (THI) values between raw and effluent coal mine water revealed a lower value in the effluent, thereby demonstrating that the treated water is cleaner than the raw water in coal mine.

### 3.2. Distribution Characteristics of Typical PTEs

Typical 11 PTEs indicators were analyzed in raw and effluent water from the upstream mining area of the Zhuozhang River. As shown in Table 1 , the average concentration of arsenic in raw mine water is 51 μg/L (range: 12–98 μg/L), which exceeds the Class III limit (10 μg·L^−1^) of the National Groundwater Quality Standards (GB/T 14848-2017) by 5.1 times. Furthermore, raw mine water from the S1 Mine was treated by chemical treatment and reverse osmosis membrane processes. The arsenic concentration in the raw mine water was reduced from 98 μg/L to 0.63 μg/L and 0.27 μg/L, with removal efficiencies as high as 99.4% and 99.7%, respectively. This demonstrates that the treatments can substantially reduce arsenic levels in polluted mine water and mitigate the associated environmental pollution.

A comparison of PTE concentrations between raw mine water and treated mine effluent showed that, with the exception of Cu and Hg, the concentrations of Pb, As, Cd, Zn, Ni, Al, Mn, Fe, and Co in the mine drainage were lower than those in the raw mine water (Table 1). Notably, among all raw mine water samples, only arsenic exceeded the Class III standard limit, while the concentrations of the other ten metal pollutants (Cu, Pb, Hg, Cd, Zn, Ni, Al, Mn, Fe, Co) were within the permissible range. In contrast, all eleven PTEs in the mine drainage were below the Class III limits of the National Groundwater Quality Standards, with no exceedances observed. This indicates that environmental supervision and management in the mining areas along the Zhuozhang River Basin are relatively effective, leading to a high compliance rate for mine drainage.

To clarify the pollution level of PTE in mine water from coal mining areas in the Zhuozhang River Basin at the national scale, this study compared and analyzed it with the PTE concentrations in mine water from other coal mines across China, with the results presented in Table 1. Compared with coal mine drainage from the Yudong River in Guizhou Province, the concentrations of Cu and Pb in the mine drainage investigated in this study were lower, while those of Cd, Zn, Ni, and Co were also significantly lower by approximately one to two orders of magnitude. Similarly, Mn, Fe, and Al were significantly lower by three to four orders of magnitude. Compared with groundwater from the Southern Jiangsu coal mine in Suzhou, the concentrations of Cu and Pb in the mine water of this study showed no statistically significant difference, whereas those of Zn, Mn, and Fe were significantly lower by approximately one to three orders of magnitude (Table 1). In comparison with raw mine water from coal mines in Inner Mongolia, Pb and As concentrations in the raw mine water of this study were at a comparable order of magnitude, while Zn, Mn, and Fe were roughly two orders of magnitude lower. By comparing with PTE concentrations in mine water from other coal mines across China, it can be concluded that the PTE pollution level of coal mine water in the Zhuozhang River Basin is relatively low (Table 1).

Further comparison between PTE levels in mine effluent and surface water within the basin demonstrated that PTE concentrations in mine effluent were 1–3 orders of magnitude lower than those in the Baisha River, Guizhou (Table 1). Relative to surface water of the Fenghe River within Shanxi Province, the ratios of PTE concentrations in mine drainage to those in Fenghe surface water were as follows: Cu (approximately 2.0), Pb (0.5), As (1.0), Cd (nearly equal), Zn (0.38), Ni (1.69), Mn (0.06), and Fe (0.03). Overall, the concentrations of several PTEs (Pb, Zn, Mn, Fe) in the mine drainage in this study were lower than those in the surface water of the Fenghe River Basin (Table 1). The reason is that the spatial variations in PTE concentrations among different basins are mainly attributed to the combined effects of geological background, pollution source composition, wastewater treatment efficiency, and hydrogeochemical conditions.

### 3.3. Mine Water Quality Evaluation

**Figure 3:**
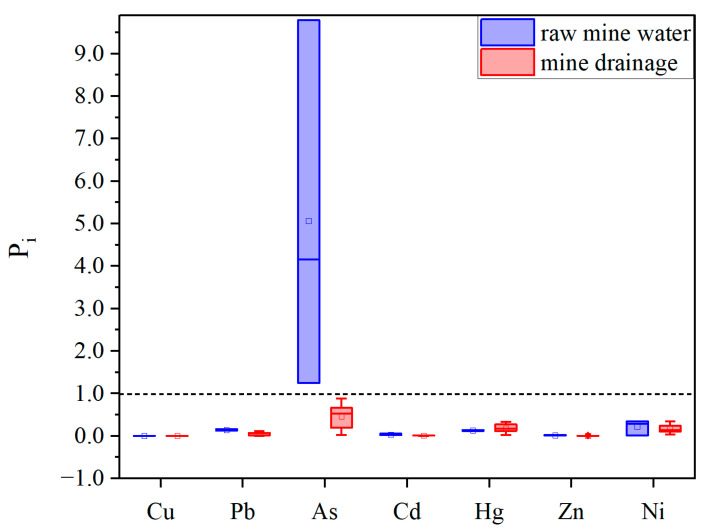
The single pollution index of PTEs in raw mine water and mine drainage in the research area. Note: The dotted line represents the position where Pi equal one.

In this study, single-factor index and comprehensive pollution index were employed to assess PTE pollution in mine water from mining areas in the upper reaches of the Zhuozhang River, with the Class III standard of the Standard for Groundwater Quality as the reference (Table S1). As presented in Table S1, water pollution levels were classified into five grades—no pollution, slight pollution, light pollution, moderate pollution, heavy pollution—based on the Pi: ≤1, 1–2, 2–3, 3–5, and >5, respectively. The results of the single-factor pollution index analysis are presented in Figure 3. As shown, analysis of Pi values of seven PTEs in raw mine water and treated mine effluent revealed that only arsenic in raw mine water had a Pi value > 1, failing to meet the Class III groundwater standard. Specifically, Pi values of arsenic in raw mine water were 9.8 (S1, severe pollution), 4.2 (S2, moderate pollution), and 1.3 (S3, slight pollution) (Figure 3). Since the Pi values of all other PTEs (Cu, Zn, Cd, Hg, Zn, and Ni) were < 1, arsenic was identified as the primary pollutant in raw mine water, which may be associated with the background As content in the mining area and hydrochemical processes of raw mine water. In addition, the order of the pollution index for PTEs in raw mine water was As > Ni > Pb > Hg > Zn > Cd > Cu, whereas in mine drainage it followed the sequence As > Hg > Ni > Pb > Cd > Zn > Cu. This discrepancy is mainly attributed to the differences in geochemical occurrence forms and the concentration of PTEs between raw mine water and mine drainage, together with the differential removal effects of conventional treatment processes on various PTEs [59].

Further comprehensive pollution index evaluation of raw mine water and treated effluent near the Zhuozhang River revealed that raw mine water at S1 had a P_N_ value of 7.0 (severe pollution), raw mine water at S2 had a P_N_ value of 3.0 (moderate pollution), and mine drainage at S2 had a P_N_ value of 0.64 (slight pollution). No PTE pollution was detected in raw mine water or mine drainage from other mining areas. In conclusion, PTE pollution in raw mine water and mine drainage in the study area is generally slight.

### 3.4. Health Risk Assessment of PTEs in Mine Water

**Figure 4:**
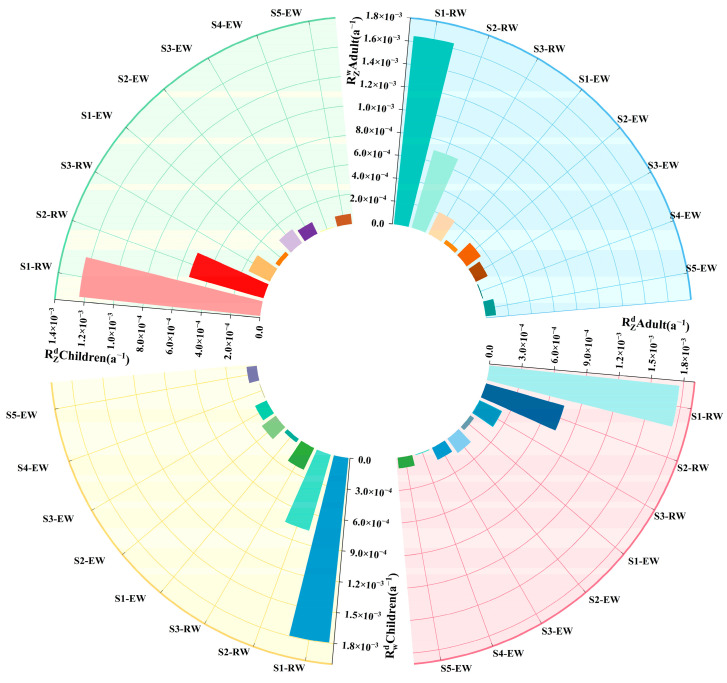
Comparison of health risks of different mine waters to adults and children via oral ingestion and dermal Contact. Note: the $R_{Z}^{w}\;$ Adults (a^–1^), $R_{Z}^{d}\;$ Adults (a^–1^), $R_{Z}^{d}\;$ Children (a^–1^), and $R_{Z}^{w}\;$ Children (a^–1^) was are shown with blue, pink, yellow, and green backgrounds, respectively.

Health risks of PTEs in water are jointly influenced by multiple factors, including the type of people and exposure pathways [60]. Based on health risk assessment models and parameters, the carcinogenic and non-carcinogenic health risks posed by PTEs in coal mine water through drinking water ingestion and dermal exposure pathways in different age groups were quantified, as presented in Figure 4. As illustrated in Figure 4, the annual health risks posed to different populations via oral ingestion and dermal contact were quantitatively compared in this study. For adults, the mean annual health risk via oral ingestion ($R_{Z}^{w}$) from raw mine water was (8.5 × 10^−4^ a^−1^) (range: (1.6 × 10^−3^ a^−1^–7.0 × 10^−4^ a^−1^), whereas the mean annual risk via dermal contact ($R_{Z}^{d}$) was 9.1 × 10^−4^ a^−1^ (range: 2.3 × 10^−4^–1.8 × 10^−3^ a^−1^). The risk associated with dermal exposure was approximately 0.6 times higher relative to oral ingestion exposure. In mine drainage, a similar pattern was observed, and the mean annual $R_{Z}^{w}$ was (7.7 × 10^−5^ a^−1^) (range: 3.7 × 10^−6^ a^−1^–1.1 × 10^−4^ a^−1^), while the mean annual $R_{Z}^{d}\;$ was (8.3 × 10^−5^ a^−1^) (range: 4.3 × 10^−6^ a^−1^–1.2 × 10^−4^ a^−1^). Thus, dermal contact presented a 0.6 times higher annual health risk for adults than oral ingestion.

For children, the mean annual $R_{Z}^{w}$ in raw mine water was 9.3 × 10^−4^ a^−1^ (range: 2.3 × 10^−4^ a^−1^–1.8 × 10^−3^ a^−1^), and the mean $R_{Z}^{d}$ was 6.4 × 10^−4^ a^−1^ (range: 5.3 × 10^−4^ a^−1^–1.2 × 10^−3^ a^−1^), representing a slight elevation in risk via oral ingestion (Figure 4). In mine drainage, the mean $R_{Z}^{w}$ was 8.4 × 10^−5^ a^−1^ (range: 4 × 10^−6^ a^−1^–1.6 × 10^−4^ a^−1^), whereas the mean annual $R_{Z}^{d}$ was 5.8 × 10^−5^ a^−1^ (range: 3 × 10^−6^ a^−1^–1.1 × 10^−4^ a^−1^). The risk posed by oral ingestion was 1.4-fold greater than that via dermal contact. (Figure 4). In summary, the results reveal that for adults exposed to both raw and treated mine water, the annual health risk from dermal contact exceeds that from oral ingestion. In contrast, oral ingestion constitutes the dominant exposure pathway for children, yielding a higher annual health risk relative to dermal contact.

The total annual health risk values (R_Z_) for raw mine water and treated mine drainage were compared, and the results are presented as follows. The Rz for adults was 1.8 × 10^−3^ a^−1^ in raw mine water versus 1.6 × 10^−4^ a^−1^ in mine drainage, while for children it was 1.6 × 10^−3^ a^−1^ and 1.4 × 10^−4^ a^−1^, respectively. Overall, raw mine water exhibited a total annual health risk approximately 1.1 times greater than mine drainage for both age groups. This confirms that children exhibit higher susceptibility to PTE exposure from mine water due to differences in physiological metabolism, behavioral patterns, and exposure dose coefficients [50]. This was mainly attributed to the higher total PTE concentration in raw mine water compared with mine drainage, as well as the high efficiency of the treatment technology applied to mine drainage. The total health risks for adults and children in the raw mine water and mine drainage both exceeded the maximum acceptable risk level (MARL), which is 5.0 × 10^−5^ a^−1^ [45].

**Figure 5:**
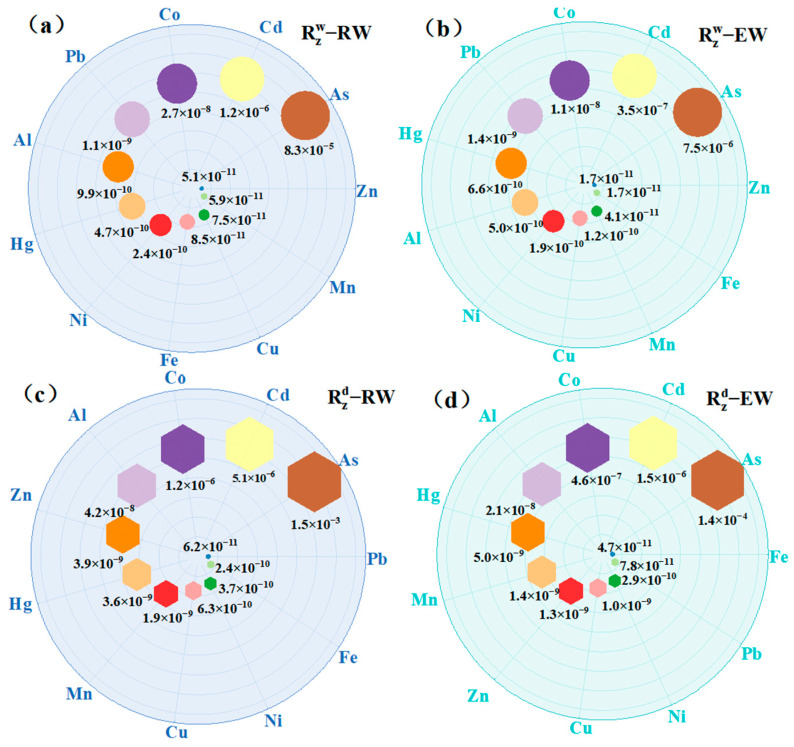
(**a**,**b**) are the $R_{Z}^{w}\;$ of PTEs in raw mine water (purple background) and mine drainage (blue background), respectively; (**c**,**d**) are the $R_{Z}^{d}$ of PTEs in raw mine water (purple background) and mine drainage (blue background), respectively. Note: $R_{Z}^{w}$ (a^−1^): per capita annual total health risks by oral ingestion resulting from PTEs, $R_{Z}^{d}$ (a^−1^): per capita annual total health risks by dermal contact pathway resulting from PTEs.

**Figure 6:**
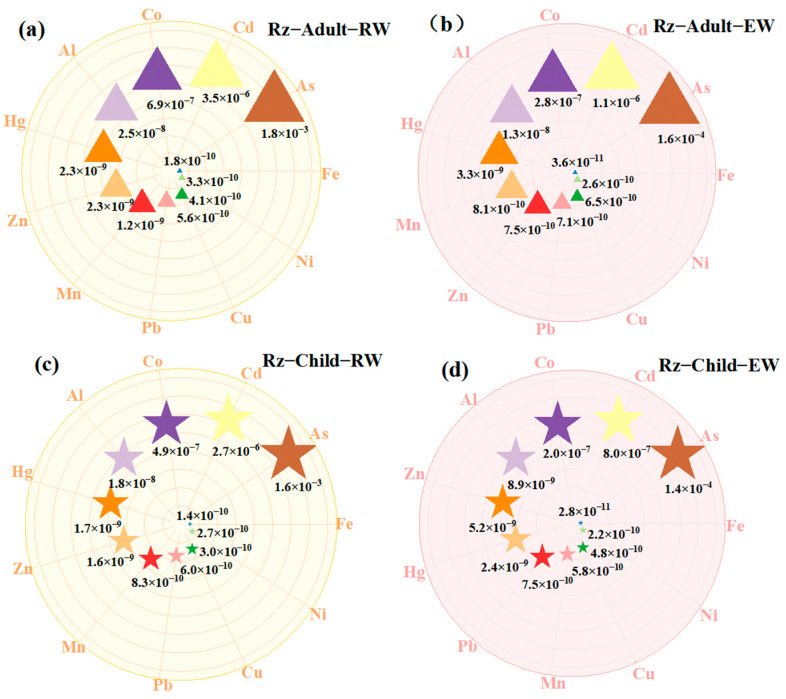
(**a**,**b**) The Rz of PTEs in raw mine water (yellow background) and mine drainage (pink background) for adults, respectively; (**c**,**d**) The Rz of PTEs in raw mine water (yellow background) and mine drainage (pink background) for children, respectively; Note: Rz (a^−1^): Per capita annual total health risks resulting from PTEs. Triangles stand for adult data, while stars stand for children’s data.

The health risks of different PTEs (HMs) in coal mine water are presented in Figure 5 and Figure 6 for adults and children, respectively. In terms of the PTE types, the health risks by the drinking pathway were in the order: As, Cd, Co, Pb, Al, Hg, Ni, Fe, Cu, Mn, Zn and As, Cd, Co, Pb, Hg, Al, Ni, Cu, Mn, Fe, Zn in the raw mine water and mine drainage. The order of health risks by the dermal contact pathway in the raw mine water and mine drainage was As > Cd > Co > Al > Zn > Hg > Mn > Cu > Ni > Fe > Pb and As > Cd > Co > Al > Hg > Mn > Zn > Cu > Ni > Pb > Fe, respectively, which was mainly related to the PTE types, concentrations, Sf, RfD, and Ed. As shown in Figure 6, the total risk Rz in raw mine water and mine drainage for adults was As > Cd > Co > Al > Hg > Zn > Mn > Pb > Cu > Ni > Fe and As > Cd > Co > Al > Hg > Mn > Zn > Pb > Cu > Ni > Fe, which the Rz of As is exceed the MARL, must be paid more attention in the risk decision-making management. The total risk Rz in raw mine water and mine drainage for children was As > Cd > Co > Al > Hg > Zn > Mn > Pb > Cu> Ni > Fe and As > Cd > Co > Al > Zn > Hg > Pb > Mn > Cu > Ni > Fe. Due to the Rz of As exceeding the MARL in the raw mine water and mine drainage, for both adults and children, more attention must be paid to risk decision-making management.

## 4. Limitation

This study systematically investigated the pollution characteristics of raw mine water and mine drainage, and further assessed their associated human health risks. Nevertheless, several limitations of this work should be acknowledged. First, the pretreatment and advanced purification processes for mine water at mining sites are largely regarded as confidential proprietary technologies. Only general categories of pretreatment methods are publicly available, while detailed operational procedures and technical parameters remain undisclosed. Accordingly, a comprehensive comparative analysis of diverse mine water treatment technologies could not be implemented in the present study. Second, raw mine water and mine effluents are rarely utilized directly as domestic drinking water; hence, this research merely serves as a fundamental exploratory investigation. Future studies can focus on in-depth exploration of pollutant source identification mechanisms and the performance evaluation of pretreatment processes, so as to enhance the practical applicability and scientific reference value of relevant research in this field.

## 5. Conclusions

This study systematically analyzed the pollution characteristics of fluoride and PTEs, along with water quality evaluation and human health risks associated with raw mine water and mine drainage in the upper Zhuozhang River Basin. Fluoride concentrations in raw mine water exceeded the WHO standards, whereas no exceedance was observed in mine drainage. Although the oral exposure risks of fluoride [61] were negligible, dermal contact risks [62] could not be ignored, and the total hazard index for all age groups was unacceptable.

For PTEs, only arsenic in raw mine water exceeded the Grade III groundwater standard, making it the primary pollutant. The discrepancy in pollution index sequences between raw and treated water was attributed to geochemical forms and the differential removal efficiency of treatment processes. Total health risks of PTEs in raw water were much higher than in drainage. Thus, long-term monitoring and targeted pollution management are essential for safe mine water utilization.

## Figures and Tables

**Table 1:** Comparison of PTE concentrations in mine water of the Zhuozhuang River with those in other rivers of China. (Note: —— = not detected).

		The Average Concentration of PTEs (μg/L)											
Sampling sites	Water sample	Cu	Pb	As	Cd	Hg	Zn	Ni	Al	Mn	Fe	Co	References
Zhuozhuang river, Shanxi, China	Raw mine water	2.6	1.4	51	0.18	0.13	14	4.3	126	7.5	23	7.5	This study
	Mine drainage	4.4	0.53	4.6	0.05	0.18	4.6	3.4	63	5.3	4.6	3.0	This study
Yudong river, Guizhou, China	Mine drainage	21	71	——	22	——	732	560	55,115	1467	287,700	230	[56]
Sunan coal mine, Suzhou, China	Ground water	1.75	0.47	0.55	——	——	31.2	——	——	266	922	——	[6]
Inner Mongolia coal mine, China	Raw mine water	——	8.1	22	——	——	3710	——	——	160	9190	——	[57]
Karst Lead–Zinc Mine, Chongqing, China	Ground water	2.95	12	0.3	31.99	——	7020.84	13.02	——	151.59	41	7.51	[45]
Baisha River, Guizhou, China	Surface water	——	——	10	——	4	180	——	9910	2750	68,480	——	[13]
Fenghe river, Shanxi, China	Surface water	2.07	0.98	1.63	0.04	——	12	2.12	——	84	144	——	[58]

## Data Availability

The original contributions presented in this study are included in the article/Supplementary Material. Further inquiries can be directed to the corresponding author.

## Supplementary Materials

The following supporting information can be downloaded at: https://www.mdpi.com/article/10.3390/toxics14070553/s1, Text S1 The detailed process of PTE human health risk assessment; Table S1 Evaluation criteria for the single-factor pollution index and the Nemerow pollution index; Table S2 Parameters for health risk assessment of fluoride in males, females, teenagers, children, and infants; Table S3 Parameters for health risk assessment of PTEs in adults and children; Table S4 Values of Pc, Sf and RfD.
