# Supplementary material for: Pollution Characteristics and Health Risk Assessment of Fluoride and Potentially Toxic Elements in Coal Mine Water of Shanxi Province, North China: A Comparative Analysis of Raw Mine Water and Mine Drainage
Source: Toxics. 2026 Jun 25;14(7):553. doi: 10.3390/toxics14070553 (PMC13417652; doi:10.3390/toxics14070553)
Supplement: Supplementary file 1 [file toxics-14-00553-s001.zip › toxics-4365357-supplementary.pdf]

Pollution Characteristics and Health Risk Assessment of Fluoride and Potentially Toxic Elements in Coal Mine Water of Shanxi Province, North China: A Comparative Analysis of Raw Mine Water and Mine Drainage

Yulu Pei [1], Jie Luo [2], Chunyu Ma [1], Pingchuan Ma [2], Xin Lin [2], Weihua Li [1], Juping Yan [2,3] and Xuejun Sun [1,4,*]

[1] School of Environmental and Resource Sciences, Shanxi University, Taiyuan 030006, China
[2] School of Environmental and Resource, Taiyuan University of Science and Technology, Taiyuan 030024, China
[3] Shanxi Key Laboratory of Coordinated Management and Control for Environmental Quality, Taiyuan University of Science and Technology, Taiyuan 030024, China
[4] Shanxi Key Laboratory of Ecological Restoration for Loess Plateau, Shanxi University, Taiyuan 030006, China
* Correspondence: sunxuejun@sxu.edu.cn

Catalog

Table S1 Evaluation criteria for the single-factor pollution index and the Nemerow pollution index 3
Table S2 Parameters for health risk assessment of fluoride in males, females, teenagers, children, and infants. 3
Text S1 The detailed process of PTE human health risk assessment............................4
Table S3 Parameters for health risk assessment of PTEs in adults and children...........6
Table S4 Values of Pc, Sf and RfD. 6

Table S1 Evaluation criteria for the single-factor pollution index and the Nemerow pollution index.

Water quality level	Pi	Pollution assessment	P_N	Pollution assessment
I	≤1	No pollution	<0.59	No pollution
II	1–2	Slightly pollution	0.59-0.74	Slightly pollution
III	2–3	Lightly pollution	0.74-1.0	Lightly pollution
IV	3–5	Moderately pollution	1.0-3.5	Moderately pollution
V	>5	Seriously pollution	>3.5	Seriously pollution

Table S2 Parameters for health risk assessment of fluoride in males, females, teenagers, children, and infants.

Intake pathway	Parameter	Male	Female	Teenagers	Children	Infants
Oral Ingestion	Ingestion Rate (L/day)	2.5	2.5	2	1	0.5
	Exposure Frequency	365	365	365	365	365
	Exposure Duration(/a)	68	70	18	12	1
	Body weight	75	60	35	20	8
	Average lifetime of the effects	24820	25550	6570	4380	365
Dermal Contact	Coefficient of skin permeability (L/m^3)	0.001	0.001	0.001	0.001	0.001
	contact time (h)	0.4	0.4	0.4	0.4	0.4
	Conversion Factor (L/cm^3)	0.001	0.001	0.001	0.001	0.001
	Average Height (cm)	175	160	150	100	75
	Absorption factor	1	1	1	1	1
	dermal contact frequency limit (L/day)	1	1	1	1	1

Text S1 The detailed process of PTE human health risk assessment

(1) Drinking pathway

Chemical carcinogenic PTEs:

$$R_w^c = \frac{Add_w \times Sf_w}{L} \quad \text{(S1)}$$

When R_w^c > 0.01,

$$R_w^c = \frac{1-\exp(-Add_w \times Sf_w)}{L} \quad \text{(S2)}$$

Chemical non-carcinogenic PTEs:

$$R_w^n = \frac{Add_w}{RfD_w \times L} \times 10^{-6} \quad \text{(S3)}$$

$$Add_w = \frac{C_a \times Ia \times Ef \times Ed}{Bw \times Ta} \quad \text{(S4)}$$

(2) Dermal contact pathway

Chemical carcinogenic PTEs:

$$R_d^c = \frac{Add_d \times Sf_d}{L} \quad \text{(S5)}$$

When R_d^c > 0.01,

$$R_d^c = \frac{1-\exp(-Add_d \times Sf_d)}{L} \quad \text{(S6)}$$

Chemical non-carcinogenic PTEs:

$$R_d^n = \frac{Add_d}{RfD_d \times L} \times 10^{-6} \quad \text{(S7)}$$

$$Add_d = \frac{Ca \times Sa \times Pc \times Et \times Ef \times Ed \times Cf}{Bw \times Ta} \quad \text{(S8)}$$

(3) Total health risk assessment model

$$R_z^w = R_w^c + R_w^n \quad \text{(S9)}$$

$$R_z^d = R_d^c + R_d^n \quad \text{(S10)}$$

$$R_z = R_z^w + R_z^d \quad \text{(S11)}$$

R_w^c, R_w^n: Per capita annual health risks (PCAHRs) by the drinking pathway resulting from chemical carcinogenic PTEs and chemical non-carcinogenic PTEs, a^{-1}.

R_d^c, R_d^n: PCAHRs by the dermal contact pathway resulting from chemical carcinogenic PTEs and chemical non-carcinogenic PTEs, a^{-1}.

R_z^w: Per capita annual total health risks by drinking pathway resulting from PTEs, a^{-1}

R_z^d: Per capita annual total health risks by dermal contact pathway resulting from PTEs,

a^{-1}.

R_z: Per capita annual total health risks resulting from PTEs.

L: Life expectancy, 70 a.

Sf_w: Carcinogenic potency factor of chemical carcinogenic PTEs by the drinking pathway, kg·d/mg.

Sf_d: Carcinogenic potency factor of chemical carcinogenic PTEs by the dermal contact pathway, kg·d/mg.

RfD_w, RfD_d: Reference dose for the daily intake of chemical non-carcinogenic PTEs by the drinking pathway and dermal contact pathway, mg/(kg·d).

Add_w: Daily exposure dose per unit of body weight of PTE by the drinking pathway, mg/(kg·d); Ca: The average PTE concentration in water, mg/L; Ef: Exposure frequency, d/a (365 d/a); Ia: Average daily water intake, L/d; Ed: Exposure duration (chemical non-carcinogenic PTEs: 35 a, chemical carcinogenic PTEs: 70 a); Bw: Body weight; Ta: Average exposure time (chemical non-carcinogenic PTEs: 12,775 d, chemical carcinogenic PTEs: 25,550 d)[1]. All the factors in the equation were presented in Table S3.

Add_d: Daily exposure dose per unit of body weight of PTE by the dermal contact pathway, mg/(kg.d); Pc: Skin penetration constant, $cm \cdot h^{-1}$; Sa: Skin contact surface area (adults: 18,000 cm^2, children: 8000 cm^2); Et: Exposure time, h d^{-1} (adults: 0.6333 h/d, children: 0.4167 h/d); Cf: Conversion factor (10^{-3} L)/(1 cm^3) = 1

Values of Pc, Sf and RfD are shown in Table S4[2].

Table S3 Parameters for health risk assessment of PTEs in adults and children.

Parameter	Meaning	Value		unit
		Adults	Children	
Ia	Average daily water intake	2.2	1	L/d
Ef	Exposure frequency	365	365	d/ a
Bw	Body weight	60	25	Kg
Sa	Skin contact surface area	18000	8000	cm^2
Et	Exposure time	0.6333	0.4167	h/d
CF	Conversion factor	1	1	mL/cm^3

Table S4 Values of Pc, Sf and RfD.

Heavy metals		Pc(cm/h)	Sf（kg.d/mg）		RfD(mg/kg.d)	
			Sf_w	Sf_d	RfD_w	RfD_d
Carinogenic PTEs	As	0.0018	32	3.66		
	Cd	0.001	6.1	6.1		
Non-carcinogenic PTEs	As	0.0018			6.5×10^{-5}	1.23×10^{-4}
	Cd	0.001			0.001	1×10^{-5}
	Cu	0.0006			0.04	0.012
	Pb	0.000004			0.0014	0.00042
	Hg	0.0018			0.0003	0.0003
	Zn	0.0006			0.3	0.001
	Al	0.01			0.14	0.14
	Mn	0.0001			0.14	0.0018
	Fe	0.0001			0.3	0.045
	Ni	0.0001			0.02	0.0054
	Co	0.01			0.0003	0.0003

References

1. Zhou, J.; Jiang, Z.; Qin, X.; Zhang, L. Heavy Metal Distribution and Health Risk Assessment in Groundwater and Surface Water of Karst Lead–Zinc Mine. *Water* **2024**, *16*, doi:10.3390/w16152179.
2. Molla, A.; Emmanouil, C.; Lolas, A.; Charvalas, G.; Kyzas, G.Z. Quantitative Health Risk Evaluation of Soil Contamination by Potentially Toxic Elements near an Industrial Area in Central Greece. *Environmental Processes* **2026**, *13*, doi:10.1007/s40710-026-00841-2.
